# Cerebral Vasospasm Pharmacological Treatment: An Update

**DOI:** 10.1155/2013/571328

**Published:** 2013-01-31

**Authors:** Ioannis Siasios, Eftychia Z. Kapsalaki, Kostas N. Fountas

**Affiliations:** ^1^Department of Neurosurgery, University Hospital of Larissa, Faculty of Medicine, University of Thessaly, Biopolis, 41110 Larissa, Greece; ^2^Department of Diagnostic Radiology, University Hospital of Larissa, Faculty of Medicine, University of Thessaly, Biopolis, 41110 Larissa, Greece; ^3^Institute of Biomolecular & Biomedical Research (BIOMED), Center for Research and Technology - Thessaly (CERETETH), 38500 Larissa, Greece

## Abstract

Aneurysmal subarachnoid hemorrhage- (aSAH-) associated vasospasm constitutes a clinicopathological entity, in which reversible vasculopathy, impaired autoregulatory function, and hypovolemia take place, and lead to the reduction of cerebral perfusion and finally ischemia. Cerebral vasospasm begins most often on the third day after the ictal event and reaches the maximum on the 5th–7th postictal days. Several therapeutic modalities have been employed for preventing or reversing cerebral vasospasm. Triple “H” therapy, balloon and chemical angioplasty with superselective intra-arterial injection of vasodilators, administration of substances like magnesium sulfate, statins, fasudil hydrochloride, erythropoietin, endothelin-1 antagonists, nitric oxide progenitors, and sildenafil, are some of the therapeutic protocols, which are currently employed for managing patients with aSAH. Intense pathophysiological mechanism research has led to the identification of various mediators of cerebral vasospasm, such as endothelium-derived, vascular smooth muscle-derived, proinflammatory mediators, cytokines and adhesion molecules, stress-induced gene activation, and platelet-derived growth factors. Oral, intravenous, or intra-arterial administration of antagonists of these mediators has been suggested for treating patients suffering a-SAH vasospam. In our current study, we attempt to summate all the available pharmacological treatment modalities for managing vasospasm.

## 1. Introduction

Aneurysmal subarachnoid hemorrhage (aSAH) constitutes a major cause of stroke, as approximately 3–15% of all stroke cases are due to ruptured intracranial aneurysms [1–4]. Data from population-based studies suggest that the incidence rates vary considerably from 6 to 20 per 100,000 population, with the highest rates reported from Japan and Finland [5–8]. Outcome after aSAH depends on several factors, including the severity of the initial event, the peri-ictal medical management, various surgical variables, and the incidence of aSAH-induced complications. Cerebral vasospasm (CV) is the most frequent and troublesome complication after aSAH.

Ecker and Riemenschneider [9] and Robertson [10] were the first ones, who pointed out the occurrence of cerebral arterial spasm following aSAH [9, 10]. Later on, Fisher and his colleagues published a synopsis regarding cerebral vasospasm [11]. Vasospasm, as the term implies, constitutes a reduction in the caliber of a vessel. However, in aSAH cases, the occurrence of vasospasm means much more than just narrowing a cerebral vessel lumen, with significant clinical ramifications. Although, cerebral vasospasm is considered a treatable clinicopathological entity, it is still responsible for many deaths and serious disabilities among patients suffering from intracranial aneurysm rupture [12–23]. The presence of cerebral vasospasm could be either clinically symptomatic or only angiographically evident. Angiographic vasospasm can be seen in up to 70% of patients with aSAH, while symptomatic vasospasm is seen in approximately 20–40% of cases [14–17, 24, 25]. Delayed Cerebral Infarction (DCI) is defined as clinically symptomatic vasospasm, or infarction attributable to vasospasm, or both, and has a peak incidence between the 4th and the 12th postictal days [26].

 The pathogenesis of cerebral vasospasm has remained poorly understood despite all recent advances in immuno-histochemistry and molecular biology. It is believed that the important role to the pathogenesis of vasospasm has the depletion of nitric oxide (NO), which is a potent vasodilator. Posthemorrhagic NO depletion has been demonstrated to cause cerebral vasoconstriction [27–30]. Other theories postulate that either the production of NO is decreased in aSAH [28, 31–33], or that the presence of extravasated hemoglobin and its degradation products may disrupt signaling between the vascular endothelium and the underlying smooth muscular layer [28, 34, 35]. This latter process induces a cascade of metabolic events, which finally leads to endothelin-1 (ET-1) production and cerebral vasoconstriction [28, 34, 35]. Endothelin-1 is a potent vasoconstrictor, which is produced in ischemia and is bound to specific receptors on smooth muscle cells causing vasoconstriction and endothelial proliferation [36–38]. It has been demonstrated that increased ET-1 levels have been found in the plasma and CSF of aSAH patients, with the presence of elevated levels of ET-1 correlating with the persistence of cerebral vasospasm [28, 39, 40]. 

Another mechanism proposed to be implicated in the development of cerebral vasospasm is the free radical oxidation of bilirubin to bilirubin oxidation products (BOXes). Bilirubin oxidation products act on vascular smooth muscle cells and stimulate vasoconstriction and vasculopathy due to smooth muscle cell injury. Data have accrued implicating BOXes in the pathogenesis of cerebral vasospasm [41]. Furthermore, CSF concentrations of BOXes correlate with the clinical occurrence of vasospasm in patients with aSAH [41, 42]. Recent data suggest that BOXes act rather by potentiating the already initiated cerebral vasospasm, than inducing cerebral vasospasm [41]. Inflammation, following subarachnoid hemorrhage, has also been postulated to play a crucial role in the pathogenesis of cerebral vasospasm [43, 44]. Cerebral vasospasm has been shown to complicate bacterial meningitis, while the nonspecific inflammation of the subarachnoid space the via injection of substances such as talc and latex beads has been shown to produce marked vascular constriction and vessel morphological changes mimicking those occurring after aSAH [43]. Inflammation and leukocyte infiltration is prominent in the cerebral blood vessel walls, following exposure to blood and its degradation products [45, 46]. Moreover, leukocyte concentrations are elevated in the CSF of patients who develop aSAH-related ischemia [47]. Leukocyte recruitment is promoted by the overexpression of adhesion molecules, which facilitate leukocyte adherence to the vascular endothelium. Indeed, adhesion molecules, such as ICAM-1, VCAM-1, and E-selectin, have been found to be elevated in the CSF of patients with aSAH and in blood vessel walls exposed to a blood clot [37, 48]. Leukocytes can contribute to vasospasm in several potential ways. They can promote free radical formation, which may evoke endothelial dysfunction and calcium influx [49, 50]. They also produce a variety of substances such as leukotrienes and ET-1, which can have powerful vascular effects [38]. Another potential mechanism by which leukocytes may contribute to vasospasm is through the consumption of nitric oxide (NO). The NO consumption has a dual effect on the development of vasospasm. Firstly, decreased concentrations of NO have as a result the weakening of its vasodilatory effects. Secondly, NO metabolism results into the formation of reactive nitrogen species, which may be toxic for the vessel wall [44]. 

Cytokines are proteins, which are powerful mediators and regulators of immune responses and have also been implicated in the pathogenesis of cerebral vasospasm following aSAH. Cytokine expression is profoundly altered following aSAH. Several cytokines have been found to be upregulated in cerebral vasospasm, including TNF-a, IL-1, IL-6, and IL-8 [51–53]. Recently, various therapies targeting cytokines have demonstrated some therapeutic efficacy in the management of cerebral vasospasm [54, 55]. 

Many controversies exist generally in the management of patients suffering aSAH and specifically in the prevention and/or the pharmacological treatment of aSAH-induced vasospasm. There have been some interesting efforts of scientific communities (American Heart Association in 2009), to create guidelines for the management of aSAH and the associated cerebral vasospasm [56]. Similarly, the Neurocritical Care Society recently published an extensive supplementary international consensus statement based on multidisciplinary expert opinion, regarding the intensive care unit management of aSAH [57]. A general consensus exists only for the oral administration of nimodipine, a well-known calcium channel blocker, to all patients suffering from aSAH [56]. It seems that nimodipine improves outcomes by decreasing the incidence of cerebral ischemia [58, 59]. The British aneurysm nimodipine trial and other clinical trials confirmed that there is a reduction in cerebral infarction in patients treated with nimodipine from 33% to 22% [60–65]. Additionally, the oral administration of nimodipine resulted in 40% reduction in poor outcomes among aSAH patients, while its administration was correlated with no major side effects [60–65]. Thus, there is class I evidence proving that oral nimodipine is definitely beneficial to aSAH patients [56]. It has to be emphasized, however, that nimodipine improves neurological outcome but not cerebral vasospasm [56]. It has been postulated that nimodipine's beneficial role may be the result of a more complex neuroprotective mechanism of action than that of its vasodilatory effect [56].

 It is evident that despite all the recent advances in microsurgical and endovascular management of aSAH, the vasospasm associated morbidity and mortality rates remain extremely and unacceptably high. The purpose of our current study is to critically review the pertinent literature regarding all pharmacological therapeutic modalities, which have been employed in the treatment of cerebral vasospasm.

## 2. Material and Methods

An extensive literature search through the PubMed medical database was performed using any possible combination of the terms “cerebral vasospasm,” “vasospasm treatment,” “aSAH,” “SAH," “delayed cerebral ischemia,” “delayed ischemic neurological deficit,” “ischemia,” “ischemic event,” “vasoconstriction,” and “aneurysm rupture". Our search was limited within the last 23 years (1990–present). All titles and abstracts identified were meticulously reviewed. Furthermore, reference lists from the retrieved articles were carefully reviewed to identify any additional pertinent articles for inclusion.

Eligible articles had English-language abstracts and were published only in peer-reviewed journals. Every possible effort was made to identify any repetition of cases among the published clinical series and/or repetition of reports in different journals. In these cases, only the original clinical series were included in our study. It has to be mentioned, however, that this task was not easy, and the reader must be aware of potential redundancies in the reported data. In reviewing the previously published clinical series, particular attention was paid to the design of each clinical study and its methodological characteristics, and special attention was paid on the pharmacological treatment and outcome of patients with aSAH and vasospasm.

## 3. Results

### 3.1. Triple “H” Therapy

For several years the employment of triple “H” therapy (hypervolemia, hypertension, and hemodilution) has widely been accepted as an efficacious treatment modality for patients suffering from aSAH, in regard to preventing or reversing vasospasm [66]. However, the existent literature provides only level B evidence regarding the utilization of triple “H” therapy in the management of patients suffering from aSAH [67].

Recently, Robinson et al. [68] reported a computational model for evaluating the actual effect of two (hypertension and hemodilution) of the three components of the triple “H” therapy. They found that in cases of severe vasospasm (narrowing more than 50% of the initial vessel diameter), the systemic blood pressure should be increased dramatically for reversing vasospasm. In addition, any decreases in the hematocrit had minimal, if any, impact on blood flow in a constricted cerebral vessel [68]. Treggiari [69] in a systematic review study showed that hypervolemia did not appear to be superior to normovolemia, while the side effects of hypervolemia were significantly more frequent. They also concluded that hypertension was associated with higher cerebral blood flow, regardless of the volume status. The latter of their findings supports the employment of hypertension in aSAH patients with delayed ischemic events [67, 69]. Meyer et al. [70] in a recent survey among the members of the Neurocritical Care Society reported that triple “H” therapy differed significantly from institution to institution, therefore the extraction of any meaningful conclusions regarding its efficacy is very difficult, if not impossible. 

Recently, MacDonald et al. [71] reported their experience from employing continuous milrinone infusion instead of triple “H” therapy. They found that milrinone regimen required less invasive monitoring and resources than triple “H” therapy, while its hemodynamic effect was comparable to that of the triple “H” therapy [67]. Further large-scale clinical trials are necessary for validating their observations. 

### 3.2. Endothelin-1 Antagonists

It is widely accepted that the interaction between ET-1 and NO is critical for maintaining adequate cerebral vascular dilatation and sufficient cerebral blood flow during aSAH. Human cerebral arteries express two types of ET-1 receptors, ETA and ETB, which evoke vasoconstriction. Numerous experimental studies have demonstrated that both ET receptor antagonists and ET converting enzyme inhibitors can reverse cerebral vasospasm, thereby support the potential role of ET in the management of aSAH-associated CV. One of the most promising pharmacological agents employed for the prevention or reversal of cerebral vasospasm is clazosentan, one of the several endothelin receptor (ETA) antagonists, which have been studied in the development of cerebral vasospasm [36]. Clazosentan has been the subject of a phase IIb clinical trial, the Clazosentan to Overcome Neurological iSChemia and Infarct Occurring after Subarachnoid hemorrhage (CONSCIOUS-1) study (Table 1). The CONSCIOUS-1 was a double-blind, randomized, clinical trial, which examined the effects of the clazosentan on cerebral vasospasm. Clazosentan decreased the incidence of severe vasospasm, the occurrence of delayed ischemic neurological deficits, and the incidence of new infarcts seen on CT scans in a dose-dependent fashion. However, it did not show a reduction in patient mortality, though the study was underpowered for this endpoint [71]. Better clinical outcome could only be found after post hoc analysis as several clinical investigators have pointed out [72–75]. 

Two phase III clinical trials, CONSCIOUS-2 and CONSCIOUS-3, have been performed in patients treated with surgical clipping or endovascular coiling [75]. In the CONSCIOUS-2 trial, patients were randomly assigned to clazosentan (5 mg/h, in 768 patients) or placebo groups (389 patients) for more than 14 days [74, 75]. The results of this study showed that clazosentan had no significant effect on mortality or CVS-related morbidity 6 weeks after aSAH (primary endpoint). Clazosentan was linked with a 17% relative risk reduction in the primary endpoint without an improvement of poor functional outcome at the 12th postictal week, as this was expressed by GOS score ≥4 (secondary endpoint). The lack of improvement according to the investigators possibly can be attributed to other pathophysiological processes than cerebral vasospasm, which contributed to the development of DCI. 

The results of the CONSCIOUS-3 trial have been recently published [76]. In a double-blinded, placebo-controlled, phase III trial, a total of 571 patients were managed (placebo group = 189 patients, clazosentan 5 mg/h = 194 patients, and clazosentan 15 mg/h = 188 patients) after endovascular coiling of their aneurysms. Poor outcome, calculated with extended GOS score <4, was occurred in 24% of the patients receiving placebo, in 25% of the patients receiving clazosentan in dosology 5 mg/h and in 28% of patients receiving high clazosentan dose. Pulmonary complications, anemia, and hypotension were observed more often in patients who received clazosentan compared to the placebo group patients. At the 12th postictal week, the observed mortality rate was 6% for the placebo group, 4% for the 5 mg/h clazosentan group, and 6% for the 15 mg/h clazosentan group. The investigators concluded that clazosentan in infusion dose of 15 mg/h reduced cerebral vasospasm, as well as the observed aSAH-associated related morbidity and mortality, without any, however, improvement of the overall clinical outcome of their participants [76].

### 3.3. Intravenous Magnesium Sulfate

Significant interest has been developed in the employment of magnesium as a vasodilatory agent for preventing and/or reversing cerebral vasospasm. Magnesium acts as a calcium antagonist and may increase cerebral blood flow by reducing vasoconstriction. Certainly, the use of magnesium has potential benefits in the setting of aSAH, with the minimal risk of severe adverse or side effects such as hypotension, hypocalcemia, or bradycardia.

#### 3.3.1. Experimental Studies

Van den Bergh et al. [110] claimed that magnesium is readily available, inexpensive, and has a well-established clinical profile in obstetrical and cardiovascular clinical practice for treating ischemia. They supported that magnesiumcan reverse delayed cerebral vasospasm and can reduce the extent of acute ischemic cerebral lesions after experimental subarachnoid hemorrhage in rats. However, MacDonald et al. in 2004 [111] tested in animals the hypothesis that a continuous intravenous infusion of Mg^++^ reduces cerebral vasospasm after experimental SAH. The authors concluded that magnesiumsulfate did not significantly reduce cerebral vasospasm after SAH in the doses tested. In 2005, Westermaier et al. [112] conducted a study to find the maximum protective dose of intravenousmagnesium sulfate in a rat model of transient focal ischemia. The authors concluded that continuousmagnesium administration for achieving stable serum concentrations between 2 and 3 mmol/L offered the best protection and was well tolerated. They extrapolated from their data that serum concentration higher than 3 mmol/L should not be attempted [112]. Recently, Sharma et al. [113] investigated, in an animal experimental study, the role of L-type Ca^++^ channels in themagnesium-induced relaxation of basilar smooth muscle cells. Their results demonstrate that L-type Ca^++^ channels are functionally expressed in rabbit basilar smooth muscle cells, and they suggest that L-type Ca^++^ channels may play a pivotal role formagnesium-induced vascular relaxation.

#### 3.3.2. Clinical Studies

In 2000, Boet and Mee [114] evaluated the effect ofmagnesiumsulfate (MgSO_4_) on the clinical course of patients with severe aSAH. They supported that a prospective, double-blinded, placebo-controlled trial was required to establish the effectiveness of MgSO_4_ for treating vasospasm in aSAH (Table 2). In 2001, Brewer et al. [77] studied the effect ofmagnesiumas a cerebral vasodilator by measuring the cerebral blood flow velocity (CBFV) response to a 5 g intravenous bolus of MgSO_4_ compared with a saline placebo, in patients with aSAH. They concluded that the administration of high-dose MgSO_4_ following aSAH is safe, while steady serum Mg^++^ levels in the range of 4 to 5.5 mg/dL could be easily maintained. They also stated that their magnesium treatment did not interfere with the patient's neurological assessment, the administration of anesthesia during surgery, or other aspects of the patient's clinical care. They observed a trend for a higher percentage of patients obtained GOS scores of 4 or 5 in the group treated with MgSO_4_, but this trend did not reach the levels of statistical significance. They also supported that a larger study was necessary for further evaluating their findings [77]. Likewise, Chia et al. [78] introduced routine magnesium supplementation in patients presented with aSAH in order to determine whether there has been a reduction in the incidence of cerebral vasospasm. The authors concluded thatmagnesiumsupplement has a beneficial role in the prevention of cerebral vasospasm following aSAH. They also supported that a larger-scale study was necessary [78]. 

In 2003, Barile et al. [79] presented a case of a 45-year-old woman who had suffered from subarachnoid hemorrhage and developed after 8 days symptomatic vasospasm in the left middle cerebral artery (MCA), while she was receiving nimodipine. Transcranial Doppler monitoring was performed. Cerebral autoregulation was abolished in the left MCA. They administered a bolus dose of magnesium sulfate, followed by a continuous intravenous infusion in order to achieve serummagnesiumlevels in the range of 4–4.5 mg/dL (administered dose = 1.65–1.85 mmol/L). Their intervention resulted in a marked decrease (12.2%) of the left MCA mean blood flow velocity, without clinically relevant change in the patient's systemic blood pressure (only 3% decrease). This effect was maintained for at least four hours. However, the temporary improvement of MCA flow velocities' and the administration of magnesium sulfate did not prevent the development of ischemic lesions in their case [79]. In 2004, Collignon et al. [80] studied the association between serummagnesiumlevels and the development of DIND, as well as the outcome of patients with aSAH. They postulated thatmagnesiumsupplementation in doses for maintaining normal or high-normal serum concentrations seems unlikely to be beneficial for vasospasm-induced DINDs [80].

At approximately the same time, Lees et al. [81] tested whetherintravenousmagnesiumsulfate, given within 12 h of stroke onset, reduces death or disability rates measured 90 days after the ictal event. Primary outcome was not improved bymagnesium administration(odds ratio = 0.95). In addition, mortality rate was slightly higher in themagnesium-treated group than that in the placebo group (hazard ratio = 1.18). Secondary endpoint analyses failed to demonstrate any magnesium treatment effect. Planned subgroup analyses showed that there was a benefit frommagnesium administrationin the incidence of noncortical strokes (*P* = 0.011), whereas a greater benefit had been expected in the cortical group. The authors concluded that magnesiumgiven within 12 h of an acute stroke does not significantly reduce the chance of death or disability, although it may be beneficial in preventing lacunar strokes [81].

The actual mechanism of the action of magnesium in patients suffering aSAH remains unclear. It has been postulated that the observed magnesium-associated diminished incidence of DINDs and ischemic events may be the result of either magnesium's neuroprotective effect or the augmentation of the cerebral blood flow by the magnesium-induced dilation of small vessels. In 2005, Chan et al. [82] evaluated the effects ofmagnesiumsulfate on brain tissue oxygen tension (PtO_2_), carbon dioxide tension (PtCO_2_), and pH in patients undergoing temporary occlusion for the clipping of a cerebral aneurysm. Data from their study suggested that magnesiumenhances tissue oxygenation and attenuates hypoxia during temporary artery occlusion [82]. Contrariwise, Prevedello et al. [83] studied magnesium sulfate as an adjuvant in the treatment of vasospasm. According to the authors, magnesiumdid not seem to interfere in vasospasm frequency but apparently acted favorably in decreasing the SAH-associated morbidity and the length of hospital stay of their patients [83]. Similarly, Yahia et al. [84] studied the safety and the feasibility of continuous high-dose intravenousmagnesiumsulfate administration for the prevention of cerebral vasospasm and ischemic cerebral injury. The authors confirmed previously published data regarding the safety and the feasibility of continuous infusion of high-dose intravenous MgSO_4_ in patients with aSAH. Likewise, Boet et al. [85] evaluated the safety and the efficacy of MgSO_4_ infusion in improving clinical outcome in patients suffering from aSAH. They concluded that MgSO_4_ infusion after aSAH is well tolerated, and it may be associated with better clinical outcome. They suggested that a larger study was required to confirm the neuroprotective effect of MgSO_4_ [85]. 

At approximately the same time, Wong et al. [86] performed a randomized, double-blind, pilot study onmagnesiumsulfate infusion for aSAH. In their small-size cohort, it was shown that intravenous MgSO_4_ infusion for aSAH seemed to be feasible. The authors suggested that a larger study recruiting approximately 800 patients was required for investigating the potential neuroprotective effect ofmagnesium in aSAH [86]. In 2006, Stippler et al. [87] investigated the effectiveness of continuous intravenous MgSO_4_ infusion for vasospasm prophylaxis. Analysis of their results suggested that MgSO_4_ infusion may have a role in the cerebral vasospasm prophylaxis, if therapy is initiated within 48 hours after the aneurysm rupture [87]. Muroi et al. [88] in a prospective, randomized, single-blinded, placebo-controlled study examined the safety and the efficacy of high-dose MgSO_4_ intravenous therapy after aSAH for preventing DIND and evaluated its impact on the patient's clinical outcome. They concluded that high-dose MgSO_4_ therapy might be efficient as a prophylactic therapy after aSAH, and it may reduce the risk of poor clinical outcome. Nevertheless, because of the observed high frequency of side effects, patients should be observed in an intensive or intermediate care setting [88].

In 2009, Zhao et al. [89], in a meta-analysis study, examined the clinical effectiveness ofmagnesiumsulfate in the prevention of cerebral vasospasm in patients suffering aSAH. They concluded that the occurrence of poor outcome (death, vegetative state, or functional dependency) in patients treated withmagnesiumsulfate was less likely than the control group patients (odds ratio = 0.54). Their observed mortality rates did not differ betweenmagnesiumsulfate (14%) and control (12%) groups (odds ratio = 1.16). Their results indicated that although there was a reduced likelihood of a poor outcome for patients treated withmagnesiumsulfate after aSAH, patient mortality was not significantly improved [89]. 

In 2010, Wong et al. [90] performed a pilot study usingmagnesiumsulfate in patients with aSAH. This phase-III study aimed to compareintravenousmagnesiumsulfate infusion with saline placebo among patients with aSAH. They concluded that their results do not support a clinical benefit from the intravenous administration ofmagnesiumsulfate [90]. Contrariwise, Westermaier et al. [91] examined whether the maintenance of elevatedmagnesiumserum concentrations by the intravenous administration ofmagnesiumsulfate can reduce the occurrence of cerebral ischemic events after aSAH in a prospective, randomized, placebo-controlled study. They concluded that high-dose intravenousmagnesiumcan reduce cerebral ischemic events by attenuating vasospasm and also by increasing the ischemic tolerance during critical cerebral hypoperfusion. Likewise, Rabinstein et al. [72] in a preliminary study for the management of patients with aSAH concluded that magnesium sulfate has potentially helpful effects in the prevention of vasospasm and delayed cerebral ischemia. On the contrary, Suarez [92], in a recent clinical study, concluded that continuous magnesium infusion is not supported by the existing published data.

Muroi et al. [93] tried to evaluate a possible link between the inflammatory response and MgSO_4_ therapy, sincemagnesium has anti-inflammatory properties. Their results indicate a suppression of inflammatory cytokine release, in particular IL-6, in patients treated with high-dose MgSO_4_. Their results call for further studies on the effect of Mg^++^ on the inflammatory signaling pathway and its influence on delayed cerebral ischemia following aSAH [92].

 Dorhout Mees et al. [94], very recently, published their results from MASH-II clinical trial. This was a phase-III randomized, placebo-controlledtrial, including eight large-volume neurovascular centers in Europe and South America. They randomly assigned (with computer-generated random numbers, with permuted blocks of four, stratified by centre) patients, aged 18 years or older, suffering from aSAH evident on brain imaging, who were admitted to the participating hospital within four days from hemorrhage. The patients were receiving intravenousmagnesium sulfate, in a dose of 64 mmol/day, or placebo. They excluded patients with renal failure or body weight lower than 50 kg. Patients, treating physicians and investigators assessing outcomes and analyzing data were masked to the allocation. The primary outcome was poor outcome or death, defined as a score of 4-5 on the modified Rankin Scale score, three months after the subarachnoid hemorrhage. A total of 1204 patients were enrolled, one of whom had his treatment allocation lost. A group of 606 patients were assigned to themagnesiumgroup (two lost to followup), while 597 to the placebo group (one lost to followup). A total of 158 patients (26.2%) had poor outcome in themagnesiumgroup compared with 151 (25.3%) in the placebo group (risk ratio = 1.03). The authors attempted in the same study to perform a meta-analysis of the previously published, prospectively obtained clinical data. Their updated meta-analysis of seven randomizedtrialsinvolving 2047 patients showed thatmagnesiumis not superior to placebo treatment for the reduction of poor outcome after aSAH (risk ratio = 0.96). They concluded that intravenousmagnesiumsulfate does not improveclinicaloutcome after aSAH; therefore, routine administration ofmagnesiumcannot be recommended based on their results and the existent literature data.

### 3.4. Statins

Statins are 3-hydroxy-3-methyl-glutaryl-CoA reductase inhibitors and it seems to have an important role in vasospasm prevention. The proposed mechanism of action for statins involves the induction of the NO pathway and the dilation of cerebral vessels, thus leading to improved cerebral blood flow [115–117]. Two small-size randomized placebo-controlled, single-center studies evaluated the safety and the feasibility of statin administration in aSAH (Table 3). In one study, pravastatin reduced the incidence of angiographically proven vasospasm and shortened the duration of severe vasospasm [95]. Another randomized, controlled trial examined the employment of simvastatin in patients suffering from aSAH [96].They demonstrated that the incidence of angiographically proven vasospasm and DIND was significantly reduced in the simvastatin group [96]. Tseng analyzed four high quality trials and concluded that statins reduce DCI and mortality rate in patients with vasospasm [118]. 

Side effect of the statins administration is the elevation of liver function, which was not significant in comparison with the placebo group in the previously performed studies. The existing data in the literature show that statins are well tolerated, with promising results [118, 119]. There is an ongoing phase-III study (STASH clinical trial), in which simvastatin is administered at a dose of 40 mg/h for 21 days versus placebo in patients with aSAH [72]. These study's results are greatly anticipated for defining the exact role of statins in the management of patients with aSAH. 

### 3.5. Fasudil

It has been reported that various protein kinases such as protein kinase C, light chain kinase, and Rho-kinase may play a critical role in the signal transduction pathway of cerebral vasospasm [120–124]. It has been demonstrated that the upregulation of Rho-kinase is observed not only in spastic arteries but also in ischemic brain tissue [120, 125, 126]. Therefore, it has been postulated that fasudil or hydroxyl-fasudil, potent Rho-kinase inhibitors, improves hemodynamic function by increasing regional cerebral blood flow. Furthermore, they may improve cerebral blood flow by limiting vascular endothelial damage and thus vascular dysfunction and also by minimizing the inflammatory response implicated in cerebral vasospasm development [120, 125–128]. 

#### 3.5.1. Experimental Studies

 Several animal experimental studies have demonstrated that intravenous or intra-arterial administration of fasudil decreases the incidence of cerebral angiographic vasospasm [120, 127, 129–132]. Liu et al. [129] showed that the administration of fasudil hydrochloride in a rabbit subarachnoid model resulted in a significant decrease in the occurrence of vasospasm. They also reported that the intra-arterial administration was more efficacious than the intravenous administration in their experimental study [129]. Likewise, Satoh et al. [128] reported on their experience from intravenously administering hydroxyl-fasudil in a canine subarachnoid hemorrhage model. They found that hydroxyl-fasudil significantly reversed vasospasm and also decreased blood viscosity in their study [128].

#### 3.5.2. Clinical Studies

Numerous clinical studies have evaluated the role of fasudil's employment in preventing or reversing cerebral vasospasm in patients suffering from aSAH [97–101, 124] (Table 4). Although Castanares-Zapatero and Hantson [73] reported that fasudil reduced the incidence and severity of vasospasm, they postulated that its administration had no significant effect on the clinical outcome of their patients. However, Iwabuchi et al. [97] found in their clinical series that the intra-arterial administration of fasudil hydrochloride resulted into the significant improvement of both, angiographic and clinical vasospasms. Similarly, Zhao et al. [98] reported that the intravenous administration of fasudil had as a result decreased the incidence of angiographic vasospasm, prevention of symptomatic vasospasm, and better clinical outcome in their cohort [98]. Likewise, Nakamura et al. [99] found that fasudil hydrochloride constitutes an effective treatment for cerebral vasospasm. They concluded that a selective intra-arterial route of administration was superior to a nonselective one [99]. Liu et al. [100] in a meta-analysis study, including eight clinical series and 843 patients, examined the efficacy of fasudil in the management of vasospasm. They found that fasudil decreased, in a statistically significant fashion, the incidence of angiographic (only 48% of that observed in the control group) and of clinical vasospasm (only 40% of that of the control group) [100]. Moreover, fasudil significantly decreased the incidence of cerebral infarction in their analysis, thus improving the overall outcome in patients suffering from aSAH [100].

The reported adverse events and side effects associated with fasudil administration may be summarized into convulsions, intracerebral hemorrhage, and blood biochemistry abnormalities [101, 133, 134]. The reported rate of blood biochemistry abnormalities (AST, ALT, *γ*-GTP, and ALP elevated values) was 15.9% in one study, and all these abnormalities were easily reversible, requiring no further treatment [101]. The convulsion rate has been reported as high as 17.4%, and its incidence seemed to be associated with the administered dose and the infusion methodology [133]. Further evaluation and reporting of any adverse events of fasudil, particularly in cases of systemic administration, is of a paramount importance for validating its long-term safety profile. Furthermore, standardization of the administered dose via selective intra-arterial infusion is necessary for establishing a widely accepted clinical practice for managing patients with aSAH.

### 3.6. Erythropoietin

A growing body of evidence has been accumulated regarding the employment of erythropoietin (EPO) in the management of cerebral vasospasm [134–141]. Although the employment of EPO has shown to decrease the occurrence of vasospasm, the exact mechanism of EPO action has remained poorly understood. Several different mechanisms such as inflammation limitation, apoptosis inhibition, oxidative damage limitation, and neurogenesis upregulation have been postulated for explaining EPO's neuroprotective action [137, 139, 141, 142]. 

#### 3.6.1. Experimental Studies

Chen et al. [134] postulated that recombinant human EPO (rhEPO) inhibited the vascular endothelial apoptosis by altering the JAK2/STAT3 signalling pathway in a rabbit SAH model. Similarly, Zhang et al. [135] worked on a rat SAH model and demonstrated that rhEPO administration activated the Nrf2-ARE pathway, and thus it modulated the cerebral oxidative stress by inducing antioxidant and detoxifying enzymes. Moreover, it has been demonstrated that the administration of high doses of EPO increases the regional brain tissue oxygen tension in patients with severe vasospasm after SAH [138].

#### 3.6.2. Clinical Studies

Tseng et al. [137] in a phase-II randomized, double-blind, placebo-controlled clinical trial evaluated the potential role of EPO intravenous administration in patients suffering from aSAH. They reported that EPO decreased the incidence of severe vasospasm in a statistically significant fashion from 27.5% to 7.5%, while the occurrence of delayed ischemic deficit was reduced from 40.0% to 7.5% [137]. They postulated that EPO reduced delayed cerebral ischemia secondary to aSAH by decreasing the frequency and severity of the observed vasospasm and also by shortening the time span of impaired cerebral autoregulation [137]. A large-scale, multi-institutional, randomized, double-blind clinical study examining the efficacy and safety of 30,000 IU EPO intravenous administration was just completed [137]. However, the results of this study have not been released yet. It is expected to provide significant information and to clarify the exact role of EPO in the management of vasospasm and prevention of delayed ischemic events in patients suffering from aSAH.

### 3.7. Nicardipine Prolonged-Release Implants

A continuously increasing number of clinical series demonstrate the safety of implanting intracisternally nicardipine prolonged-release pellets and its effectiveness in preventing or reversing cerebral vasospasm [102–109, 143] (Table 5). Kasuya et al. [102] employed nicardipine prolonged-release implants (NPRIs) in the CSF cisterns of patients with severe aSAH and thick blot clots in the subarachnoid space (Fisher grade 3 SAH). They found that the usage of NPRIs reduced the occurrence of delayed ischemic neurological deficits from 11% in the control group to 6% [102]. Barth and his coworkers [103] reported that the incidence of angiographic vasospasm in their single-institution series decreased from 73% in the control group to only 7% in the NPRIs group. Furthermore, the incidence of CT-proven delayed ischemic lesions was reduced from 47% to 14%, while the modified Rankin and the National Institute of Health Stroke scale scores were significantly better in the NPRIs group in their cohort [103]. They reported that the employment of NPRIs decreased the mortality rate from 38% in their control group to only 6% in the NPRIs group [103].

Similarly, Krischek et al. [104] reported that the employment of NPRIs decreased the incidence of vasospasm and the occurrence of cerebral infarcts in their series. They noted, however, that the effect of NPRIs is more potent in the proximal parts of arteries, while it becomes less evident in the distal to the implantation parts [104]. Barth et al. [105] investigated the effect of NPRIs on the functional outcome and the quality of life of patients suffering from aSAH, one year after the initial ictal event. They found that the Karnofsky Performance Scale and the Mini-Mental Status Examination scores were significantly better among patients treated with NPRIs. However, the SF-36 and the Hamilton Depression Rating Scale scores were similar among patients with NPRIs and their control group [105]. Schneider et al. [106] examined the effect of NPRIs in the development of cerebral vasospasm. They found that the employment of NPRIs reduced the incidence of angiographic vasospasm from 44% observed in the surgically clipped control group to 11%, while the respective percentage among their patients receiving endovascular coiling was 48%. Their incidence of permanent cerebral infarctions was only 7% in the NPRIs group, compared to 22% among patients surgically clipped but having no NPRIs and 28% among patients undergoing coiling [106]. Likewise, Thomé et al. [107] postulated that the intracisternal usage of NPRIs decreased the incidence of angiographic vasospasm from 70% to only 10%.

The recent increased employment of endovascular coiling treatment in patients with aSAH allows for the development of a novel implantation strategy for nicardipine pellets in order to manage cerebral vasospasm. Indeed, Barth and his coinvestigators [108] recently reported the implantation of NPRIs in the ventricular system of patients undergoing coiling procedures for ruptured aneurysms. They found that NPRIs intraventricular implantation had no problems, and no adverse events or side effects were detected in their cohort. The monitored intracranial and cerebral perfusion pressures, heart rate, and mean arterial pressure were not affected by NPRIs, while the incidence of posthemorrhagic hydrocephalus was not any higher among patients with NPRIs [108]. The delivered dose may be increased, and the long-term effect in the incidence of angiographic vasospasm and the occurrence of ischemic events remain to be evaluated in large-scale randomized, double-blinded, clinical studies. Additionally, the effect of intraventricularly implanted that nicardipine theoretically may have a more global effect than the intracisternally delivered, a scenario that needs to be investigated in the future.

### 3.8. Sildenafil Citrate

It has been demonstrated that guanylate cyclase is actively involved in the relaxation of the vascular smooth muscle cells by converting guanosine triphosphate to cyclic guanosine monophosphate (cGMP) in the endothelial cells of the cerebral arteries [109, 144]. The intracellular accumulation of cGMP results into smooth muscle relaxation, while the decreased concentration of cGMP causes vasoconstriction [144, 145]. It is also well known that the concentration of cGMP is affected by the phosphodiesterase isoenzyme type V (PDE V), which hydrolyzes cGMP and thus causes vasoconstriction [144, 145]. Therefore, it has been postulated that the administration of PDE V inhibitors may be a useful tool in the prevention of vasospasm.

#### 3.8.1. Experimental Studies

 Inoha et al. [145] administered sildenafil citrate, which is a PDE V inhibitor, in a canine SAH model. They observed that sildenafil citrate caused partial relaxation of the vasospastic basilar canine artery in their experimental in vivo study. They concluded that PDE V inhibitors, such as sildenafil, may have a role in the prevention of cerebral vasospasm [145]. Likewise, Atalay and his coinvestigators [144] employed sildenafil citrate in a rabbit SAH model. They reported a significant vasodilatory effect for sildenafil citrate, which was observed in both normal and vasospastic rabbit cerebral vessels. However, they noted no effect caused by sildenafil in the endothelial apoptosis process [144]. Gokce et al. [146] studied the effect of sildenafil citrate in cerebral vasospasm in a rat SAH model. They found that the administration of sildenafil caused the vasodilation of the cerebral arteries and also altered the levels of lipid peroxidation, without affecting though the endothelial apoptosis process. They suggested that the employment of sildenafil in association with an apoptosis-blocking agent may be beneficial for preventing or reversing cerebral vasospasm, and it deserves to be further tested [146]. 

### 3.9. Nitric Oxide Progenitors

It has been demonstrated that nitric oxide (NO) constitutes a potent endogenous vasodilator, which directly acts on vascular smooth cells causing vascular relaxation [147]. It also has been shown that the presence of hemoglobin and its degradation products disrupt signalling between the vascular endothelium and the underlying smooth muscular layer [28, 147–150]. Thus the altered NO production represents an undoubtful pathophysiologic mechanism implicated in the pathogenesis of cerebral vasospasm in aSAH [126, 147]. It has been postulated that the administration of NO could theoretically have a direct vasodilatory effect in spastic cerebral vessels. However, the NO has an extremely short half-life, mandating thus the administration of other substances which may act as NO donors [126]. Unfortunately, many of these substances are strong and nonselective vasodilators, and when they are systemically administered, they cause significant hypotension [126]. 

#### 3.9.1. Experimental Studies

Several investigators have experimented with other than systemic (selective intra-arterial, intrathecal, and/or intracisternal, or inhalational) routes of administration of NO donors with varying results [98, 126, 150–152]. Furthermore, different NO donors, such as sodium nitrite (NaNO_2_), have been utilized in a primate SAH model and healthy volunteer human studies with an excellent safety profile and promising vasodilatory efficacy [126, 152, 153]. Recently, Osuka et al. [152] reported on their experience from employing adiponectin in a rat SAH model. They found that adiponectin concentration was significantly elevated in the CSF after the induction of SAH, thus causing a significant cerebral artery vasodilation. They proposed, as the most probable mechanism of action for adiponectin, the activation of AMP-activated protein kinase *α* and endothelial nitric oxide synthase signalling pathway [154]. Pradilla and his coinvestigators [153] reported their experience from systemically administering L-citrulline in an Hp 2-2 mouse SAH model. They examined in their study the safety of L-citrulline, as well as its effect on basilar artery spasm, on the neurobehavioral scores of the experimental animals, and on the expression of endothelial NO synthase (eNOS). They found that L-citrulline was safe and increased the patency of the basilar artery. Similarly, the neurobehavioral scores were improved after the administration of L-citrulline, and the eNOS expression was also increased [153]. Their findings, although very promising, definitely require a further validation before designing a human study.

### 3.10. Various Pharmacological Agents

A very limited number of human studies provide information regarding the employment of dantrolene in patients with cerebral vasospasm after aSAH [154–156]. Majidi et al. [154] reported an aSAH case in which they administered dantrolene via a microcatheter selectively in the spastic cerebral arteries. Interestingly, they confirmed the vasodilatory effect of dantrolene without noticing, however, its systemic hypotensive action [152]. Muehlschlegel and her coworkers [155] reported a limited case series in which they administered dantrolene intravenously, and they observed improved transcranial Doppler artery velocities. They concluded that dantrolene had a vasodilatory effect in cerebral arteries, most possibly via an intracellular smooth muscle calcium influx through a ryanodine calcium channel [155]. A few years later, Muehlschlegel et al. [156] reported the results of their pilot clinical study, in which they administered a single dose of dantrolene in patients with aneurysmal SAH. They noted that dantrolene in their cohort attenuated vasoconstriction. However, they also noticed that dantrolene may lower the systemic blood pressure [156]. 

It has been previously postulated that other pathophysiologic mechanisms than vasospasm may contribute in post-SAH morbidity and mortality [125, 149, 157]. Inflammatory mechanisms, endothelial apoptosis, cortical spreading depolarization, microthrombosis, and lipid peroxidation have been implicated in the pathogenesis of delayed ischemic events [125, 157]. Therefore, the administration of a potent anti-inflammatory agent may play a neuroprotective role in patients with aSAH. Trehalose, a nonreducing disaccharide, has strong anti-inflammatory actions [158]. Echigo et al. [158] investigated the role of intracisternal trehalose in a rabbit SAH model. They found that trehalose produced an inhibitory effect on vasospasm, in addition to its anti-inflammatory action and its effects on the lipid peroxidation process [158]. Further animal studies are necessary for accurately evaluating the role of trehalose or other similar agents in the management of SAH. Likewise, Rasmussen et al. [159] have initiated a pilot clinical study examining the effects of continuous low-dose prostacyclin infusion on regional blood flow and cerebral vasospasm, the results of which are expected with a great interest. 

## 4. Conclusions

The development of cerebral vasospasm remains the most common and troublesome complication of aSAH. It is still responsible for keeping the aSAH-associated morbidity and mortality unacceptably high, despite all recent advances in vascular microsurgery and endovascular techniques. In addition, the exact pathophysiological mechanism or mechanisms responsible for vasospasm remain still ill-defined. Although several treatment strategies have been proposed for managing cerebral vasospasm, none of them seem to efficiently and universally work. The existence of so many different treatment modalities, the different schemes and dosologies of even the same treatment strategy, and the significant variation of the utilized outcome measures, makes often the comparison of all these treatment options impossible.

In our current study, we attempt to summate the existent literature on each of the commonly employed pharmacological treatment modalities and provide an update of the most recent publications on each pharmacological category. There is a significant amount of evidence in the literature supporting the neuroprotective role of nimodipine in patients with aSAH. This is not the case, however, with all other treatment options available for vasospasm. The employment of triple “H” therapy seems not to be supported by the published so far data, and the current trend is to maintain a normo-volemic status for the vast majority of patients with aSAH. Even in those cases that ischemic events are likely, induced hypertension does not seem to be efficient in reversing vasospasm, based on the recent data of novel computational models. However, newer pharmacological agents, such as milrinone, have a safer profile and may well be of some benefit in maintaining adequate cerebral blood flow. Needless to mention that further testing is required for validating these promising but preliminary results.

The administration of ET-1 antagonists has been proven to cause no significant improvement in the clinical outcome of patients with aSAH. Similarly, the systemic administration of magnesium cannot be generally recommended based on the currently published data. The potential role of magnesium in relieving the persistent SAH-associated headache needs to be explored in the future.

In regard to the employment of statins in the management of vasospasm, the currently available data have shown that in the majority of cases there is a definite vasodilatory effect on the cerebral vasculature. The results of the STASH trial are expected to clarify the exact effect of statins in the clinical outcome of patients with aSAH.

The results of fasudil's employment in patients suffering from aSAH are controversial. Additionally, the indicated dosage for optimal results has not been standardized, although there is emerging evidence that the selective intra-arterial administration is the preferred route of administration. Further clinical studies are mandatory before recommending fasudil's routine clinical application. Likewise, the usage of erythropoietin has produced promising results with a significant decrease of vasospasm incidence and some improvement of cerebral autoregulation, as well as improved oxidative stress handling capacity. However, the expected results of the currently ongoing clinical trials may define EPO's role in the treatment of patients suffering from aSAH.

The role of NPRIs remains to be defined, since the initial experience with them was very encouraging. The concept of NPRIs intraventricular implantation becomes very appealing, in the era of the continuously expanding endovascular treatment of intracranial aneurysms. The safety profile of intraventricular implantation, along with nicardipine's vasodilatory and neuroprotective effects, requires an undoubtedly further exploration. Furthermore, several animal studies have documented the vasodilatory effect of sildenafil in spastic cerebral arteries. However, its systemic side effects, its actual vasodilatory effect in human cerebral vasculature, and its overall safety profile have to be thoroughly investigated. Likewise, the administration of newer nitric oxide progenitors in aSAH patients has produced quite promising results, while their systemic side effects are less frequent and less profound. These substances along with new pharmacological agents with anti-inflammatory properties and actions may become valid treatment options for cerebral vasospasm. The importance of performing large-scale, multi-institutional, randomized clinical trials, along with the usage of unified outcome criteria in these trials, are of a paramount importance for establishing general guidelines for vasospasm management.

## Figures and Tables

**Table 1:** Summary of clinical data regarding ET-1 antagonists in managing cerebral vasospasm.

Authors/year of publication	Type of study	Number of patients	Results of the study
Macdonald et al. (2008) [71] (CONSCIOUS-1)	Randomized, double-blind, placebo-controlled phase-II dose-finding trial	413 patients	Reduction of vasospasm in a dose-dependent fashion from 66% in the placebo group to 23% in the 15 mg/h clazosentan group (risk reduction, 65%; 95% CI, 47% to 78%; *P* < 0.0001) Clazosentan was associated with pulmonary complications, hypotension, and anemia

Macdonald et al. (2011) [74] (CONSCIOUS-2)	Randomised, double-blind, placebo-controlled phase-III trial	Clazosentan group (5 mg/h, *n* = 768 patients) and placebo group (*n* = 389 patients)	The primary endpoint was met in 161 patients (21%) of the clazosentan group and in 97 patients (25%) of the placebo group. Relative risk reduction 17%, (95% CI −4 to 33; *P* = 0.10). Poor functional outcome (GOSE score ≤ 4) occurred in 224 (29%) in the clazosentan group and in 95 patients (25%) in the placebo group. Mortality was 6% in both groups. Clazosentan was associated with lung complications, anaemia, and hypotension

Macdonald et al. (2012) [76] (CONSCIOUS-3)	Randomized, double-blind, placebo-controlled, phase-III trial	CONSCIOUS-3 was halted prematurely following the completion of the CONSCIOUS-2. A total of 577/1500 of planned patients were enrolled, and 571 were treated (placebo, *n* = 189 patients; clazosentan 5 mg/h, *n* = 194 patients; clazosentan 15 mg/h, *n* = 188 patients).	The primary endpoint was reached in 50/189 of placebo-treated patients (27%), compared with 47/194 patients (24%) treated with clazosentan 5 mg/h and 28/188 patients (15%) treated with clazosentan 15 mg/h. Poor outcome (extended Glasgow Outcome Scale score ≤ 4) occurred in 24% of patients in the placebo group, 25% of patients with clazosentan 5 mg/h, and 28% of patients with clazosentan 15 mg/h. Mortality was 6%, 4%, and 6% with placebo, clazosentan 5 mg/h, and clazosentan 15 mg/h, respectively. Clazosentan-associated complications were pulmonary complications, anemia, and hypotension

**Table 2:** Summary of clinical data regarding magnesium sulphate in managing cerebral vasospasm.

Authors/year of publication	Type of study	Number of patients	Results
Brewer et al. (2001) [77]	Prospective	14 patients (11 women, 3 men; mean age: 58 years)	Four patients developed cerebral vasospasm. Doubling serum magnesium levels did not affect MCA CBFV but slightly lowered mean arterial blood pressure and systemic vascular resistance. Intravenous magnesium bolus did not reduce elevated CBFV in the subset of SAH patients with clinical vasospasm.

Chia et al. (2002) [78]	Prospective	23 patients divided into 2 groups. An Mg^++^ receiving and a control group	7 out of 10 patients who did not receive magnesium developed vasospasm requiring intra-arterial papaverine, compared with only 2 of 13 patients among the Mg^++^ group *P* < 0.008).

Barile et al. (2003) [79]	Case report	1 patient	Continuous infusion in order to achieve serum magnesium levels in the range of 4–4.5 mg/dL (1.65–1.85 mmol/L) resulted in a marked decrease (12.2%) of the left MCA mean blood flow velocity.

Collignon et al. (2004) [80]	Prospective	128 consecutive patients	There was no significant difference in mean, minimum, or maximum serum magnesium levels between patients with and without DIND (1.93, 1.83, and 2.02 versus 1.91, 1.84, and 1.97 mg/dL, resp.,). Similarly, no difference was found in mean serum magnesium levels among patients with severe (1.94 mg/dL), moderate (1.92 mg/dL), or no DIND (1.91 mg/dL).

Lees et al. (2004) [81]	Randomized, placebo-controlled study	2589 patients	Primary outcome was not improved by magnesium (odds ratio 0.95, 95% CI 0.80–1.13, *P* = 0.59). Mortality was slightly higher in the magnesium-treated group than in the placebo group (hazard ratio 1.18, 95% CI 0.97–1.42, *P* = 0.098). Secondary outcomes did not show any treatment effect.

Chan et al. (2005) [82]	Randomized, prospective study	18 patients	Magnesium infusion increased PtO_2_ by 34%. Following temporary artery occlusion, PtO_2_ and pH decreased and PtCO_2_ increased in both groups. However, tissue hypoxia was less severe, and the rate of PtO_2_ decline was slower in the magnesium group.

Prevedello et al. (2006) [83]	Prospective	72 patients, group A placebo, group B Mg^++^	In group A, vasospasm was correlated with a longer hospitalization time, different from group B, in which patients with vasospasm receiving magnesium sulfate required less hospitalization time (*P* = 0.0003).

Yahia et al. (2005) [84]	Prospective, pilot study	19 patients (mean age: 55 years; range: 39–84 years; 11 males, 8 females)	Vasospasm was observed in 9 patients (by clinical examination in two, TCD in five, and angiogram in nine). The mean serum Mg level was 2.7 mM/L and was maintained during the infusion period. No clinical adverse effects, hemodynamic changes, or fluctuations in serum glucose or phenytoin. None of the patients died. No CT evidence of ischemic infarction. Most patients had good outcomes (GOS 5 in 10 patients; GOS 4 in 8 patients).

Boet et al. (2005) [85]	Randomized, double-blind study	45 patients receive either MgSO_4_ 80 mmol/day or saline infusion for 14 days	Patients receiving MgSO_4_ seemed to have fewer neurological deficits, better functional recovery, and an improved outcome score. However, none of these outcome variables reached a statistical significance. The incidence of cardiac and pulmonary complications in the MgSO_4_ group (43%) was also similar to that in the saline group (59%).

Wong et al. (2006) [86]	Randomized, double-blind, pilot study	60 patients receive either MgSO_4_ 80 mmol/day or saline infusion for 14 days. Patients also received intravenous nimodipine	The incidence of symptomatic vasospasm decreased from 43% in the saline group to 23% in patients receiving MgSO_4_ infusion, but it did not reach statistical significance, (*P* = 0.06). For patients who had transcranial Doppler-detected vasospasm, the duration was shorter in the magnesium group compared with controls (*P* < 0.01). There was, however, no difference between groups in functional recovery or outcome.

Stippler et al. (2006) [87]	Comparative matched-cohort study	Seventy-six adults (mean age 54.6 years; 71% women; 92% Caucasian)	Symptomatic vasospasm was present at a significantly lower frequency in patients who received MgSO_4_ infusion (18%) compared with patients who did not receive MgSO_4_ (42%) (*P* = 0.025). There was no significant difference in mortality rate at discharge (*P* = 0.328). A trend toward improved outcome as measured by the modifed Rankin Scale (*P* = 0.084), but not the Glasgow Outcome Scale (*P* = 1.0), was seen in the MgSO_4_ treated group.

Muroi et al. (2008) [88]	Prospective, randomized, single-blind, placebo-controlled study	58 patients; 27 received placebo and 31 MgSO_4_	The difference in the occurrence of DIND and secondary infarction was not significant. The intention-to-treat analysis revealed a trend toward a better outcome (*P* = 0.083) after 3 months. On-treatment analysis showed a significantly better outcome after 3 months (*P* = 0.017) and a trend toward a better outcome after 1 year (*P* = 0.083). Significantly more often hypotension (*P* = 0.040) and hypocalcemia (*P* = 0.005) occurred as side effects in the MgSO_4_ group. In 16 patients (52%), the MgSO_4_ therapy had to be stopped before day 12 because of side effects.

Zhao et al. (2009) [89]	Meta-analysis study	Five previously published clinical studies	The occurrence of poor outcome (death, vegetative state, or dependency) in patients treated with magnesium sulfate was less likely than control group patients (odds ratio (OR) 0.54 (95% confidence interval, CI 0.36–0.81)). Mortality rates did not differ between magnesium sulfate (14%) and control treated (12%) patients (OR 1.16 (95% CI 0.51–2.65)).

Wong et al. (2010) [90]	Prospective, randomized, placebo-controlled study	Of the 327 patients recruited, 169 were randomized to receive treatment with i.v. MgSO_4_ and 158 to receive saline (placebo)	The results do not support a clinical benefit from intravenous magnesium sulfate infusion.

Westermaier et al. (2010) [91]	Prospective, randomized, placebo-controlled study	110 patients receive i.v. MgSO_4_ or placebo	The incidence of delayed ischemic infarction was significantly lower in magnesium-treated patients (22% versus 51%; *P* = 0.002); 34 of 54 magnesium patients and 27 of 53 control patients reached a good outcome (*P* = 0.209). Delayed ischemic neurologic deficit was reduced (9 of 54 versus 15 of 53 patients; *P* = 0.149) and transcranial Doppler-detected vasospasm was significantly reduced in the magnesium group (36 of 54 versus 45 of 53 patients; *P* = 0.028).

Suarez (2011) [92]	Review study	Seventeen articles were identified and reviewed, including one phase-III randomized-controlled clinical trial and six phase-II randomized-controlled trials	Due to inconsistently reported benefits and the occurrence of side effects, phase-II data suggested that intravenous magnesium for SAH provided either no overall net benefit or uncertain tradeoffs. Benefit was likewise not supported in the single phase-III clinical trial.

Muroi et al. (2012) [93]	Prospective study	15 patients. Eight patients were treated with standard therapy alone (group 1) and seven patients were treated with an additional, high-dose of MgSO_4_ (group 2)	Serum Mg^++^ levels in group 2 were significantly higher compared to group 1: 1.48 ± 0.04 mmol/L versus 0.90 ± 0.01 mmol/L, *P* < 0.001. Interleukin-6 (IL-6) in the CSF was significantly lower in group 2 compared to group 1: 6680 ± 989 versus 11079 ± 1277 pg/mL, *P* = 0.021. A trend towards lower systemic IL-6 levels was found in group 2: 58 ± 7 versus 104 ± 21 pg/mL, *P* = 0.052. Systemic IL-1*β* levels were significantly lower in group 2: 0.66 ± 0.11 and 0.15 ± 0.01 pg/mL (*P* < 0.001), while the CSF levels did not differ.

Mees et al. (2012) [94]	Phase-III randomized, placebo-controlled	1204 patients—606 patients were assigned to the magnesium group (two lost to followup) and 597 to the placebo group (one lost to followup)	158 patients (26.2%) had poor outcome in the magnesium group compared with 151 (25.3%) in the placebo group (risk ratio (RR) 1.03, 95% CI 0.85–1.25). Their updated meta-analysis of seven randomized trials involving 2047 patients shows that magnesium is not superior to placebo for the reduction of poor outcome after aneurysmal subarachnoid haemorrhage (RR 0.96, 95% CI 0.86–1.08).

**Table 3:** Summary of clinical data regarding statins in the management of cerebral vasospasm.

Authors/year of publication	Type of study	Number of patients	Results
Tseng et al. (2005) [95]	Randomized prospective study	80 patients	The incidence of vasospasm and severe vasospasm were reduced by 32% (*P* = 0.006) and 42% (*P* = 0.044), respectively. The duration of severe vasospasm was shortened by 0.8 days (*P* = 0.068) in the pravastatin group. The duration of impaired autoregulation was shortened bilaterally (*P* < or = 0.01), and the incidence of vasospasm-related DINDs and mortality were decreased by 83% (*P* < 0.001) and 75% (*P* = 0.037), respectively, in the pravastatin group.

Lynch et al. (2005) [96]	Randomized prospective study	39 patients were randomized to receive either simvastatin (80 mg daily; *n* = 19) or placebo (*n* = 20) for 14 days.	The highest mean middle cerebral artery transcranial Doppler velocities were significantly lower in the simvastatin-treated group (103 ± 41 versus 149 ± 47; *P* < 0.01). In addition, vasospasm was significantly reduced (*P* < 0.05) in the simvastatin-treated group (5 of 19) compared with those receiving placebo. No patients developed clinical symptoms of myopathy or hepatitis. von Willebrand factor and S100beta were decreased in patients receiving simvastatin.

**Table 4:** Summary of clinical data regarding fasudil in the management of cerebral vasospasm.

Authors/year of publication	Type of study	Number of patients	Results
Iwabuchi et al. (2011) [97]	Retrospective study	90 cases; one placebo group and a fasudil group	Fasudil improved angiographic and clinical vasospasm.

Zhao et al. (2011) [98]	Randomized, open trial	63 patients received fasudil and 66 received nimodipine	The clinical outcomes were more favourable in the fasudil group than in the nimodipine group (*P* = 0.040). The proportion of patients with a good clinical outcome was 74.5% (41/55) in the fasudil group and 61.7% (37/60) in the nimodipine group

Nakamura et al. (2013) [99]	Prospective study	A total of 31 patients. 10 patients received nonselective intra-arterial infusion, while 10 others selective. Eleven patients were in the placebo group.	By univariate linear regression analysis, intra-arterial infusion score negatively correlated with CT score (*P* = 0.016), but was significantly correlated with GOS (*P* = 0.035).

Liu et al. (2012) [100]	Review study	8 studies—843 patients	The incidence rate of symptomatic and angiographically proven vasospasm in the fasudil group was only 48% of that of the placebo group.

Enomoto et al. (2010) [101]	Retrospective analysis	23 patients undergoing intra-arterial infusion of fasudil hydrochloride in 49 vessels	Intra-arterial fasudil infusion was effective for treating cerebral vasospasm. Infusion at a constant rate of 3 mg/min delivered by infusion pump improved the symptoms of cerebral vasospasm.

**Table 5:** Summary of clinical data regarding nicardipine prolonged release implants (NPRIs) in the management of cerebral vasospasm.

Authors/Year of Publication	Type of study	Number of patients	Results
Kasuya et al. (2005) [102]	Prospective study	97 patients among 125 surgically clipped patients	Four (6%) of the 69 patients treated with NPRIs and 3 (11%) of the 28 patients not treated with NPRIs developed delayed ischemic neurological deficits (DINDs). Of these patients, clinical deterioration with infarction occurred in two patients (3%). Eighty-six patients (89%) had an independent status at three months.

Barth et al. (2007) [103]	Prospective study	32 patients	The incidence of angiographic vasospasm in proximal vessel segments was significantly reduced after implantation of NPRIs (73% in the control group versus 7% in the NPRIs group). CT scans revealed a lower incidence of delayed ischemic lesions (47% in the control versus 14% in the NPRIs group). The NPRI group demonstrated more favourable modified Rankin and National Institute of Health Stroke scales scores, as well as a significantly lower incidence of deaths (38% in the control versus 6% in the NPRIs group).

Krischek et al. (2007) [104]	Prospective study	100 patients	Only seven patients developed DIND and five patients suffered cerebral infarction. Angiography performed on days 7–12 revealed no vasospasm in any of the arteries close to the site of NPRI placement. NPRI placement can completely prevent vasospasm.

Barth et al. (2009) [105]	Retrospective study	18 patients	Functional outcome improvement is associated with NPRIs. Also administration of NPRIs resulted into: lower incidence of cerebral vasospasm, decreased incidence of new infarcts, and lower morbidity.

Schneider et al. (2011) [106]	Prospective controlled study	81 patients	The incidence of vasospasm was 48%, 44% and 11% for patients after endovascular treatment, microsurgical clipping without NPRIs, and microsurgical clipping with NPRIs, respectively. New cerebral infarctions occurred in 28%, 22% and 7% of their groups, respectively. A good clinical recovery 1 year after was seen in 48%, 50% and 77% of their patients, respectively.

Thomé et al. (2011) [107]	Review study	Unknown	Several clinical protocols revealed that NPRIs dramatically reduce the incidence and severity of angiographic vasospasm. They found that NPRIs resulted into reduction of cerebral infarctions, and delayed ischemic neurologic deficits. On average, the incidence of angiographic vasospasm decreased from approximately 70% to less than 10%. Efficacy seemed to be dose-dependent.

Barth et al. (2011) [108]	Prospective study	31 patients	A significant positive angiographic effect caused by NPRIs could only be observed in surgically clipped patients.

Kasuya (2013) [109]	Prospective study	Unknown	Vasospasm was completely prevented in cerebral arteries by placing NPRIs adjacent to them intraoperatively. No complications were reported.
